# Einsamkeitserfahrungen junger Menschen – nicht nur in Zeiten der Pandemie

**DOI:** 10.1007/s12592-022-00415-7

**Published:** 2022-05-30

**Authors:** Severine Thomas

**Affiliations:** Institut für Sozial- und Organisationspädagogik, Universität Hildesheim, Universitätsplatz 1, L 144, 31141 Hildesheim, Deutschland

**Keywords:** Einsamkeit, Teilhabebericht der Bundesregierung, Jugendforschung, COVID-19, JuCo-Studien, Loneliness, Participation Report of the German government, Youth research, COVID-19, JuCo studies

## Abstract

Inden vergangenen Jahren hat das Thema Einsamkeit an Raum im wissenschaftlichen und politischen Diskurs gewonnen. So gibt es inzwischen zahlreiche Forschungsarbeiten und Publikationen zu Einsamkeit im Alter, die auch in der öffentlichen und politischen Diskussion besonders wahrgenommen werden. Empirische Befunde sollen diese Wahrnehmung unterstützen. Anhand von Daten des Sozioökonomischen Panels (SOEP) aus dem Jahr 2016 unterstreicht der Dritte Teilhabebericht der Bundesregierung, dass sich 16 % der Befragten oft einsam fühlen, unter den Menschen mit Beeinträchtigungen sind es sogar 31 %. Seit der Corona-Pandemie wird aufgrund der angeordneten Regelungen zum Social Distancing eine Häufung von Einsamkeitserfahrungen festgestellt. Eine Studie des wissenschaftlichen Dienstes der EU-Kommission kam zu dem Ergebnis, dass 25 % der EU-Bürger:innen sich nach den ersten Monaten der Pandemie einsam fühlten (im Vergleich zu 12 % im Jahr 2016. Regelmäßige Befragung zu Familie, Erwerbsarbeit, Politik und u. a. Gesundheit mit ca. 15.000 teilnehmenden Haushalten bzw. 30.000 Personen.). In nordeuropäischen Ländern hat sich der Anteil der einsamen Menschen seit Beginn der Pandemie sogar vervierfacht. Nach und nach etabliert sich dabei die Erkenntnis, dass Einsamkeit auch unter jungen Menschen kein seltenes Phänomen ist, das aber in dieser Altersgruppe in der Pandemie überdurchschnittlich an Bedeutung gewinnt. Dieser Beitrag analysiert Einsamkeitserfahrungen junger Menschen insbesondere anhand der Forschungsergebnisse aus den Studien JuCo I–III zu der Lebenssituation und den Erfahrungen von Jugendlichen und jungen Erwachsenen während der Corona-Pandemie und diskutiert abschließend, welcher Forschungsbedarf sich daraus ableiten lässt.

## Abstract (en)

In recent years, the topic of loneliness has gained significance in scientific and political discourses. Thus, there are now numerous research papers and publications on loneliness in old age, which are also particularly perceived in the public and political discussion. Empirical findings are intended to support this perception. Based on data from the Socioeconomic Panel (SOEP) from 2016, the Third Participation Report of the German government underlines that 16% of the respondents often feel lonely and among people with impairments it is even 31%. Since the coronavirus pandemic an increase in experiences of loneliness has been noted due to mandated regulations on social distancing. A study by the EU Commission’s Scientific Service concluded that 25% of EU citizens felt lonely after the first months of the pandemic compared to 12% in 2016 (regular survey of family, gainful employment, politics and health, with about 15,000 participating households or 30,000 persons). In northern European countries the proportion of lonely people has even quadrupled since the beginning of the pandemic. Gradually, the realization is becoming established that loneliness is not a rare phenomenon even among young people but it is gaining above average importance in this age group during the pandemic. This paper analyzes experiences of loneliness of young people especially based on the research results from the studies JuCo I–III on the living situation and experiences of adolescents and young adults during the corona pandemic and concludes by discussing which further research needs can be derived from this.

## Einsamkeit – eine begriffliche Einordnung

In der Einsamkeitsforschung wird zwischen sozialer Isolation, Alleinsein und Einsamkeit unterschieden. Soziale Isolation wird als eine Dimension verstanden, mit der die soziale Eingebundenheit von Menschen objektiv beschrieben wird, z. B. anhand der Qualität, Häufigkeit und Zahl sozialer Kontakte. So werden für die Abschätzung sozialer Vereinzelung in Gesellschaften Indikatoren wie die Zahl der Single-Haushalte oder der unverheirateten Erwachsenen herangezogen (Luhmann und Bücker 2019). Bei den Haushaltstypen kann es sich allerdings um bewusst gewählte Lebensformen handeln, sodass diese Größen nur im Kontext weiterer Aspekte für die Identifikation von sozialen Ausschlüssen und Vereinzelungen herangezogen werden können. Für Einzelpersonen gelten Indikatoren wie die Dauer und Häufigkeit von sozialen Kontakten oder die Größe der sozialen Netzwerke als Bezugsgrößen für die Bestimmung von sozialer Isolation (Tesch-Römer und Huxhold 2019).

Einsamkeit beschreibt die subjektive Erfahrung, die mit sozialer Isolation einhergehen kann. Sie gilt als eine individuell erlebte Diskrepanz zwischen den vorhandenen und gewünschten sozialen Beziehungen (Luhmann 2021; Russell et al. 2012; Peplau und Perlmann 1982). Es haben sich psychologische Skalen, insbesondere die UCLA Loneliness Scale (Russell 1996) und die De Jong Gierveld Loneliness Scale (De Jong Gierveld und van Tilburg 2010) etabliert, die das Erleben von Einsamkeit anhand vorgegebener Kriterien abbilden sollen. Auch eine Skala explizit für das Erleben junger Menschen wird dabei herangezogen (Loneliness and Aloneness Scale for Children and Adolescents (LACA) vgl. Loades et al. 2020). Dabei wird nicht direkt nach Einsamkeit gefragt, sondern anhand von Aspekten wie Häufigkeit von Kontakten oder Umfang des sozialen Netzwerks auf das Vorliegen von Einsamkeitserfahrungen geschlossen. Bisher liegen darüber hinaus kaum strukturelle oder lebenslagenbezogene Modelle vor, die die sozioökonomische Entstehung bzw. Bedingung von Einsamkeitserfahrungen beschreiben.

Einsamkeit geht mit negativen, belastenden Gefühlen einher (Petrich 2011), wohingegen Alleinsein nicht zwangsläufig zu Einsamkeitsgefühlen führen muss. Es kann eine Wahlentscheidung sein – sich temporär oder ggf. für eine längere Zeit in eine Phase des Alleinseins zu begeben. Für das Entstehen und eine mögliche Verfestigung von Einsamkeit ist relevant, dass Menschen vorübergehend oder dauerhaft ihre sozialen Bedürfnisse nach Kontakt und Zugehörigkeit nicht erfüllen können. Hier sind für die Betroffenen grundsätzlich eher die Qualität als die Quantität der sozialen Beziehungen entscheidend für das Erleben von Einsamkeit (Hawkley et al. 2008). Einsamkeit ist aber nicht nur ein individuelles Empfinden und somit als psychisches Merkmal einzustufen, sondern entfaltet sich in einem sozialen Gefüge und in der alltäglichen (reduzierten) Interaktion, die insbesondere von verfügbaren sozioökonomischen Ressourcen abhängt. Daher wird Einsamkeit neben den psychologischen Fachdiskursen insbesondere auch in der Armuts- und Ungleichheitsforschung thematisiert (Böhnke 2008; Böhnke und Link 2018).

Vor diesem Hintergrund kann das Phänomen Einsamkeit auch als Ausdruck einer Lebenslage verstanden werden, die sich in einem mangelnden Zugang zu sozialen Ressourcen und gesellschaftlichen Infrastrukturen ausdrückt. Somit lässt sich Einsamkeit als mangelnde soziale Teilhabe und fehlenden Möglichkeiten der Befriedigung eigener sozialer Bedürfnisse (Russel et al. 2012) und somit als Gegenbild zu einer inklusiven Gesellschaft einordnen.

Weitere gesellschaftliche Entwicklungen wie die soziale Ausgrenzung von ganzen gesellschaftlichen Gruppierungen oder die Verschärfung von sozialen Ungleichheiten und fehlenden Teilhabechancen (Deutscher Bundestag 2020) tragen dazu bei, dass Menschen sich nicht gut eingebunden fühlen und in verlässlichen sozialen Milieus bewegen (Dörre 2020). Inzwischen verdichten sich die Forschungsbefunde, dass die Betroffenheit von Einsamkeit nicht kontinuierlich mit dem steigenden Lebensalter wächst, sondern, dass es unterschiedliche vulnerable Lebensphasen und -bedingungen (Bundesministerium für Arbeit und Soziales 2021) gibt. Besonders gefährdete Gruppen sind junge Erwachsene und die Ältesten unter den älteren Menschen (Baarck et al. 2021; Neu und Müller 2020). Dennoch liegen bisher zu Einsamkeit im Jugend- und jungen Erwachsenenalter nur wenige Forschungsbefunde vor. Neben einschlägige Arbeiten aus der psychologischen, psychiatrischen und medizinischen Forschung (Loades et al. 2020) u. a. über Prädiktoren für das Erleben von Einsamkeit gibt es bisher wenig Erkenntnisse über das strukturelle Zusammenwirken von Einsamkeit und sozialen Exklusionsprozessen und der Verschärfung von sozialen Ungleichheiten unter jungen Menschen.

## Einsamkeit im Jugendalter – Offene Forschungsfragen

Individualisierung und Vereinzelung in modernen Gesellschaften wirkt sich auf die Lebenswirklichkeit von jungen Menschen aus. Sie wird zwar durch eine institutionalisierte Kindheit und Jugend geprägt (Bock et al. 2020), somit also frühzeitig neben der Einbettung in ihre häuslichen Lebenswelten in soziale Interaktionsarmut mit Peers oder pädagogischen Fachkräften in mehr oder weniger öffentliche Kontexte eingebunden, gleichzeitig sind die informellen sozialen Beziehungen und Gemeinschaften jedoch diverser geworden und stellen sich weniger selbstverständlich her. Gerade angesichts der digitalen Formen sozialer Teilhabe stehen analoge soziale Beziehungen nicht immer oder nicht nur in der unmittelbaren Lebenswelt zur Verfügung, sondern müssen aktiver gesucht, ausgehandelt und individuell aufrechterhalten werden. Gemeinschaftliche Wohn- und Lebensformen oder quartierbezogene soziale Netzwerke können von jungen Menschen nicht leicht erschlossen werden, aber auch die institutionellen sozialen Kontexte, z. B. in Schulen, gewährleisten keinen Schutz vor Einsamkeit. In sozialräumlichen Vergleichen zeigt sich, dass Einsamkeitserfahrungen zwischen Wohngebieten mit unterschiedlichen sozioökonomischen Merkmalen ungleich ausgeprägt sind (Bundestransferstelle Sozialer Zusammenhalt 2021). Strukturelle Aspekte von Einsamkeitserfahrungen junger Menschen werden aber bisher kaum in wissenschaftlichen Studien erhoben und systematisch ausgewertet. Viele bisherige Untersuchungen von Einsamkeitserfahrungen im Jugendalter arbeiten mit Skalen aus der Psychologie, Kinder- und Jugendpsychiatrie oder dem Public Health (Lim et al. 2019; Lee et al. 2020), die in Deutschland auf psychometrische Arbeiten und die deutsche Neukonstruktion einer Einsamkeitsskala von Bortz und Döring (1993) aufbauen.

Da junge Menschen in der Regel in familiären Kontexten aufwachsen und sich in Betreuungs- und Bildungsinstitutionen bewegen, wird das Phänomen von Einsamkeit im Kindes- und Jugendalter kaum vermutet. Es wird allenfalls als Randerscheinung wahrgenommen und nicht als verbreitetes Phänomen unter jungen Menschen identifiziert. Die digitalen Lebenswelten, in denen sich viele junge Menschen selbstverständlich bewegen, werden als sozialer Raum und Möglichkeit zu globaler Vernetzung interpretiert, seltener auch als Gefahr sozialer Vereinsamung aufgefasst (Twenge et al. 2021). Auch mit Beginn der Pandemie lag zunächst der Fokus auf älteren Menschen und den Folgen, die die Distanzregelungen und Isolierung in den Pflegeheimen sowie das Abstandsgebot auch außerhalb von Institutionen für ältere Menschen psychosozial nach sich ziehen könne. Die öffentliche Berichterstattung stärkt diesen Eindruck, obwohl bereits im Juni 2020 in einer Veröffentlichung des Sozioökonomischen Panels unterstrichen wird, dass Einsamkeitserfahrungen vor der Pandemie über alles Altersgruppen relativ gleich verteilt waren, abgesehen von Menschen mit einem direkten Migrationshintergrund (Entringer et al. 2020). Während der Pandemie haben in der Umfrage des Panels vor allem jüngere Menschen angegeben, sich besonders einsam zu fühlen und unter den fehlenden Möglichkeiten, sozialen Beziehungen pflegen können, zu leiden (ebd.).

**Figure 1:**
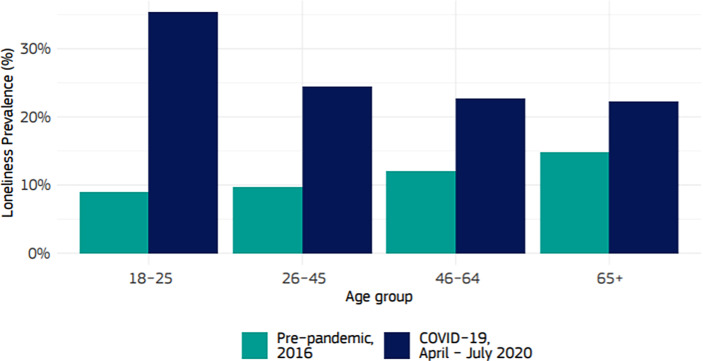

Die wissenschaftliche Forschung über Einsamkeitserfahrung bekräftigt, dass sich Menschen in höherem Lebensalter seit der Pandemie häufiger einsam fühlen (vgl. u. a. Huxhold und Tesch-Römer 2021) und bestätigt damit das öffentliche Bewusstsein. Seit Beginn der Corona-Pandemie kommen wissenschaftliche Studien zu dem Ergebnis, dass sich insbesondere unter jungen Menschen die Einsamkeitserfahrungen und psychischen Belastungen verstärkt haben (Andresen et al. 2020a, 2021; Neu und Müller 2020; Schlack et al. 2020; Schools Health and Wellbeing Improvement Research Network 2020). Die bereits genannte Studie der EU-Kommission stellt einen Vergleich unterschiedlicher Altersgruppen her: Gegenüber 2016 (9 % aller Befragten im EQLS Survey^1^) fühlten sich im Frühjahr/Sommer 2020 35 % der 18- bis 25-jährigen Teilnehmenden des LWC Survey einsam (Baarck et al. 2021, S. 21).^2^ Aktuelle Daten des Sozioökonomischen Panels weisen ebenfalls für Deutschland junge Menschen unter 30 Jahren als die Gruppe aus, die am stärksten unter Einsamkeit, Angst und Depressionen während der Pandemie leiden (Entringer und Kröger 2021; Abb. 1).

Es entwickelt sich in Anfängen ein sozialpolitischer Diskurs, u. a. aus der Armuts- und Ungleichheitsforschung (vgl. u. a. Refaeli und Achdut 2020) oder der Urbanistik (Bundestransferstelle Sozialer Zusammenhalt 2021) bzw. der Wirtschaftsforschung (D’Hombres et al. 2021; Eyerund und Orth 2019) heraus, welcher soziale Kontakte und die Qualität sozialer Beziehungen als Ausdruck hinreichender sozioökonomischer Ressourcen markiert und dabei auch junge Menschen besonders in den Blick nimmt. Demzufolge lässt sich ein Zusammenhang von Einsamkeitserfahrungen und einzelnen Risikofaktoren identifizieren, die eine individualisierte, auf das psychosoziale Erleben reduzierte Betrachtung allein als nicht sinnvoll erscheinen lassen. Schließlich unterstreichen Forschungsergebnisse, dass Einsamkeit ein Risiko für soziale Teilhabe, Gesundheit oder die Bewältigung sozialer Problemlagen darstellt (Lelkes 2013). Es stellt somit ein gesellschaftspolitisches Thema dar, welches über psychologische Perspektiven auf Einsamkeit hinausweist und soziostrukturelle Entstehensbedingungen in den Blick rückt.

## Einsamkeitserfahrungen junger Menschen unter dem Einfluss der Corona-Pandemie – Ergebnisse der JuCo-Studien

Mit den Befunden aus verschiedenen Jugendstudien, die während der Pandemie durchgeführt wurden, lässt sich herausarbeiten, dass Einsamkeit während dieser gesellschaftlichen Krise zu einer der gravierendsten sozialen Erfahrungen junger Menschen geworden ist (Andresen et al. 2020b, 2021, 2022; Entringer und Kröger 2021; Lippke et al. 2021; Reis et al. 2021; Langmeyer et al. 2020). Das Abgeschnittensein von vertrauten sozialen Netzwerken und Institutionen wie der Schule, dem Sport und Freizeitstätten, informellen Gelegenheiten für sozialen Kontakt und Austausch, hat gezeigt, dass junge Menschen diese sozialen Ressourcen für ihre Entwicklung, ihren Schutz und auch ihr Wohlbefinden besonders benötigen. Sie konnten ihre sozialen Kontakte nicht mehr alltäglich pflegen oder neue erschließen, Angehörige nicht treffen oder kaum Hilfen in Krisen in Anspruch nehmen. Ausgeprägt war unter jungen Menschen das Gefühl, nicht gehört zu werden, anfangs insbesondere auch durch vertraute Institutionen wie die Schule (Andresen et al. 2020a), aber auch durch Vertreter:innen aus Politik (Andresen et al. 2020a, b, c, 2022). Dies hat Ohnmachtsgefühle und Hilflosigkeit hervorgerufen.

Dennoch rücken junge Menschen, das wird seit in den Fachdiskursen und auch der Medienberichterstattung sehr deutlich, stets verzögert mit ihren pandemiebedingten sozialen, emotionalen und psychischen Belastungen (Angst vor Ansteckung, Einsamkeitserfahrung, fehlendes politisches Gehör, mangelnder Kinderschutz, unklare Impfstrategie für jüngere Menschen etc.) in den Blickpunkt. Seit Beginn der Pandemie haben sich allerdings die Lebenswelten von Familien gravierend verändert. Vor allem junge Menschen wurden in ihren Möglichkeiten sozialer Teilhabe, so z. B. in der Pflege ihrer Peerbeziehung, Hobbies oder der Präsenz im öffentlichen Raum erheblich eingeschränkt. Der Weg in die Institutionen, die sie normalerweise aufsuchen (Schule, Freizeitstätten, Sportanlagen etc.) war nicht zugänglich oder es gab erhebliche Einschränkungen aufgrund von Hygieneregeln – sozialen Verboten – insbesondere im öffentlichen Raum (Heyer et al. 2022; Pananese und Azzarita 2021; Voigts 2020), aber auch die Zahl der direkten Kontakte wurde für den privaten Raum reglementiert. Der Haushalt/die Wohnung wurde zur Kerneinheit sozialen Lebens. Das Zusammensein mit Eltern, Geschwistern oder anderen Personen hat mehr Raum eingenommen. Für alleinlebende junge Menschen hingegen entstand eine Lebenswelt mit nur wenigen Kontaktmöglichkeiten in Präsenz. Dies ist für viele junge Menschen eine neue Lebenserfahrung in einer neuen gesellschaftlichen Ordnung mit großer Tragweite in die privaten Lebenswelten hinein. Die Entwicklung von Einsamkeitserfahrungen unter jungen Menschen während dieser Zeitspanne werden nachfolgend anhand einzelner Befunde der JuCo-Studien, die im Frühjahr 2020, Herbst 2020 und Herbst 2021 durch den Forschungsverbund Kindheit – Jugend – Familie in der Corona Zeit der Universitäten Frankfurt und Hildesheim durchgeführt wurden, genauer analysiert.

### Methodisches Vorgehen, Sampling und Datengrundlage

Ziel der Online-Befragung war, die Lebenssituation und das Erleben junger Menschen in den Monaten der Pandemie genauer zu beschreiben. Aus rechtlichen und forschungsethischen Gründen richtete sich die Befragung explizit an junge Menschen ab 15 Jahren, denn Teilnehmende müssen einwilligungsfähig sein und die Tragweite der Einwilligung überschauen können (Verbände der Markt- und Sozialforschung Deutschland 2021). Die Erhebung orientierte sich an bereits mehrfach validierten Fragen und Skalen der Studie „Children’s Worlds+“ (Andresen et al. 2019) zu Aspekten des Wellbeing und an einem Konzept von Bedarfsdimensionen (ebd.). Es wurden subjektive Einschätzungen zur Zufriedenheit in verschiedenen Lebensbereichen, z. B. mit dem Homeschooling oder der Vertretung jugendlicher Interessen durch die Politik, zum Vorhandensein von Bezugspersonen, die ansprechbar sind, Ausstattungsmerkmale und die finanzielle Situation oder die Verfügbarkeit eines ungestörten Raums abgefragt (Wilmes et al. 2020). Die an die gegenüber Children’s World+ ältere Zielgruppe angepassten Skalen wurden um Fragestellungen zur Pandemie ergänzt (z. B. zur Sorge um die Infektion mit dem Corona-Virus). Die Reihe der JuCo-Studien lehnen sich, ähnlich wie Children’s Worlds+ und andere Studien, an das multidimensionale Konzept des Wellbeing, hinsichtlich der subjektiven Seite des Erlebens als auch der objektiven Lebenslagen und -bedingungen (Ben-Arieh et al. 2014; Andresen et al. 2019; Rees et al. 2020) an, also fokussieren die Möglichkeiten einer förderlichen Lebenswelt, um persönliche, soziale und persönliche Ziele umzusetzen. Mit dem Konzept des Wel-Being können so auch die Bedarfe und Teilhabemöglichkeiten junger Menschen im Zusammenspiel mit ihren Lebenslagen und den vorhandenen Ressourcen erfasst und Ungleichheiten festgestellt werden.

Der Fragebogen wurde bei allen Erhebungen der JuCo-Studien über Sosci Survey (https://www.soscisurvey.de/) online gestellt. Die Laufzeiten waren jeweils etwa 14 Tage. Der Fragebogen der JuCo-Studie enthielt verschiedene Dimensionen, die ebenfalls in anderen Studien zum Alltagserleben, zu Ungleichheit und zum Wohlbefinden eingesetzt werden. Sozioökonomische Daten wie Haushaltsgröße, Fragen zur Familienform, Erwerbstätigkeit und Ressourcen, Fragen zur aktuellen Zufriedenheit in verschiedenen Bereichen sowie Erfahrungen mit Bildungseinrichtungen wurden hier einbezogen. Zentral waren zudem Fragen nach den aktuellen Erfahrungswelten zu Hause, der Umgang mit Kontakten und die Zufriedenheit damit, die Wahrnehmung der verbrachten Zeit vor der Pandemie und währenddessen.

Die individuellen Möglichkeiten, mit den veränderten Alltagswellten umzugehen, hingen sehr von den Lebensumständen junger Menschen ab. Durch Fragen zu den Wohnverhältnissen, den existenziellen Voraussetzungen für das Bestreiten der neuen Lebenssituation, der Möglichkeiten zur Kommunikation und Aufrechterhaltung von Freundschaften sowie darüber, wie gut die jungen Menschen sich über Corona-Maßnahmen, Risiken etwa der Infektion, über Entscheidungen von Schulen, Universitäten und Arbeitgebern etc. informiert fühlen, sollte eine differenzierte Betrachtung ermöglicht werden (Wilmes et al. 2020). Auch wurde explizit nach Sorgen der jungen Menschen gefragt. Im Frühjahr 2020 lag der Fokus auf den Beschränkungen des sozialen Lebens aufgrund des erstmalig verhängten Lockdowns.

In allen Erhebungen wurde die Möglichkeit gegeben, Rückmeldungen zum Fragebogen zu geben. Diese sind jeweils in die Entwicklung der nachfolgenden Fragebögen eingeflossen. So wurde in JuCo II zusätzliche Fragen zur Freizeitgestaltung und in JuCo III zu dem Erleben in unterschiedlichen Phasen der Pandemie sowie zum Impfen integriert. Auch eine genauere Analyse der emotionalen Auswirkungen der Pandemie wurde durch erweiterte Fragebatterien möglich. So wurde in der Befragung im Rahmen von JuCo I deutlich, dass bereits im Frühjahr zahlreiche junge Menschen von Einsamkeitsgefühlen betroffen waren. Dies kam insbesondere in den Freitextfeldern, die am Ende des standardisierten Fragebogens zur freien Äußerung zur Verfügung standen, zum Ausdruck. Auf diesen Befund hin wurde in JuCo II auch im standardisierten Teil explizit nach Einsamkeitserfahrungen gefragt, die damit auch quantitativ erfasst werden konnten.

Als Stichprobenverfahren wurde ein Snowball-Sampling angewandt (Gabler 1992). Private und berufliche Kontakte wurden genutzt, um jungen Menschen auf die Befragung aufmerksam zu machen. Auch Einrichtungen und Fachkräfte der Kinder- und Jugendhilfe sowie der Jugendarbeit wurden als Multiplikator:innen genutzt. Die Datensätze von JuCo I bis III wurden folgendermaßen bereinigt. Es wurden nur Teilnehmende berücksichtigt, die

- die angegeben haben, zwischen 15 und 30 Jahren alt zu sein,
- die die letzte Seite der Befragung erreicht hatten und
- von denen mindestens 90 % des Fragebogens ausgefüllt wurden.

Insgesamt haben an den Studien in den sehr kurzen Erhebungszeiträumen sehr viele junge Menschen teilgenommen (Tab. 1).

**Table 1:** 

	JuCo I April/Mai 2020	JuCo II November 2020	JuCo III Dezember 2021
*n (bereinigter Datensatz)*	5520 Personen	7038 Personen	6159 Personen
*Durchschnittsalter*	19,04 Jahre	19,61 Jahre	20,0 Jahre
*Geschlecht*	65,8 % weiblich;	66,9 % weiblich	70,0 % weiblich
	31,6 % männlich	31,7 % männlich	26,8 % männlich
	2,6 % divers	1,4 % divers	2,9 % divers
*Aktuelle Beschäftigung*	56,6 % Schüler*innen	40,8 % Schüler*innen	31,2 % Schüler*innen
	18,3 % Studierende	23,2 % Studierende	24,0 % Studierende
	11,1 % Erwerbstätige	12,3 % Erwerbstätige	21,2 % im FWD
	7,2 % in Ausbildung	10,5 % im FWD	11,8 % Erwerbstätige
	2,8 % im FWD	7,6 % in Ausbildung	7,6 % in Ausbildung

*FWD* Freiwilligendienst

Der Fragebogen wurde bei den Erhebungen KiCo II und III auch in einfacher Sprache angeboten und die Verteilerwege erweitert. Dadurch sollten weitere Zielgruppen (z. B. junge Geflüchtete, junge Menschen mit Beeinträchtigungen) besser angesprochen werden.

### Einsamkeitserfahrungen junger Menschen während der Pandemie – Ergebnisse der qualitativen und quantitativen Daten der JuCo-Studien

In der Studie JuCo I war über die große Teilnehmer:innenzahl hinaus bereits besonders auffallend, dass die Aussagen zu Einsamkeit von vielen Studienteilnehmer:innen unaufgefordert in den Freitextfeldern erteilt wurden. In der ersten Studie wurde im quantitativen Teil nicht explizit danach gefragt. Für manche haben sich diese Erfahrungen in Zeiten der Pandemie verschärft, für andere wurden sie erst durch die Kontaktregelungen und Pandemiemaßnahmen hervorgebracht. Dies wird im Folgenden mit zwei Aussagen junger Menschen aus den Freitexten der Datenerhebung illustriert:

Zitat 1

Das schlimmste Übel seit der Corona-Pandemie ist für mich, dass ich keine Möglichkeit mehr habe meine beste Freundin zu treffen. Sie wohnt mit dem Zug eine Stunde entfernt bei ihrer Mutter und dessen Ehemann. Beide sind Risikopatienten und gehen zudem ziemlich hysterisch mit dem Thema Corona um. Ich habe sie nun seit März nur einmal sehen können. Das lässt mich einsam fühlen und macht mich unzufrieden mit meinem Leben. (JuCo I)

Zitat 2

Der schwierigste Aspekt ist für mich persönlich die soziale Distanzierung zu Freunden und Familie. Die Verwehrung von physischem Kontakt und dem persönlichen Treffen vieler Freunde (besonders gleichzeitig) macht mir sehr zu schaffen. Es ist eine Herausforderung mit dieser Einsamkeit und Distanz klarzukommen. Jedoch sehe ich den Sinn in den Regelungen und der Distanzierung und möchte daher auch keine Risiken eingehen! Auch wenn man dafür eventuell bis an die eigenen Schmerzgrenzen bezüglich Empfindungen wie z. B. Einsamkeit stößt. Je disziplinierter wir alle uns an die Regelungen halten und uns in Verzicht üben, desto schneller wird uns der Kontakt zu vielen wieder möglich sein. (JuCo II)

Mit der Durchführung der weiteren Studien wurde aufgrund der qualitativen Befunde aus JuCo I eine Frage zu Einsamkeit ergänzt. Auf einer Fünferskala (stimme gar nicht zu/stimme zu/teils-teils/stimme zu/stimme voll zu) konnten die Studienteilnehmer:innen angeben, ob sie sich einsam fühlen. An diesen Ergebnissen kann weiterhin gezeigt werden, dass Einsamkeit nicht nur unter alleinlebenden jungen Menschen vorkommt, auch junge Menschen, die mit ihren Familien oder andere Personen zusammenleben, stimmen der Aussage „Ich fühle mich einsam“ zu (Tab. 2).

**Table 2:** 

Ich fühle mich einsam							
	Wohnsituation						
	Mit Familie	Pflegefamilie	Jugend-WG betreutes Wohnen	Allein	Private WG	Mit Partner:in	Sample gesamt
*n* JuCo II	4716	27	90	609	782	650	7016
*n* JuCo III	3706	23	82	578	687	662	5857
*Stimme nicht zu*							
JuCo II	41,2 %	40,7 %	36,7 %	19,7 %	32,6 %	54,2 %	39,3 %
JuCo III	38,8 %	43,5 %	36,8 %	29,1 %	36,1 %	53,4 %	38,8 %
*Teils/teils*							
JuCo II	25,3 %	11,1 %	24,4 %	25,5 %	26,2 %	26,0 %	25,5 %
JuCo III	25,4 %	21,7 %	23,3 %	24,2 %	23,3 %	23,0 %	24,7 %
*Stimme zu*							
JuCo II	33,5 %	48,1 %	38,9 %	54,8 %	41,2 %	19,8 %	35,2 %
JuCo III	36,0 %	34,8 %	46,3 %	46,7 %	40,6 %	23,5 %	36,5 %

An diesen Befunden zeigt sich, dass unter allen Studienteilnehmer*innen der Befragung JuCo II im November 2020 (*n* = 7038) 35,2 % der Aussage zustimmen „Ich fühle mich einsam“. Von den jungen Menschen, die in Familien leben, stimmen nur geringfügig weniger als im Gesamtsample (33,5 %) dieser Aussage zu. Das bedeutet, dass sich ein Drittel der jungen Menschen, die mit ihren Familien zusammenleben, sich innerhalb dieses sozialen Umfelds einsam fühlen. Auch auf 41,2 % junger Menschen, die in WGs leben, trifft dies zu. Besonders ausgeprägt sind allerdings die Gefühle von Einsamkeit unter den Alleinlebenden: 54,8 % in dieser Teilgruppe befürworten die Aussage *Ich fühle mich einsam*. Am wenigsten einsam fühlten sich die jungen Menschen, die mit Partner:in zusammenleben (19,8 %). In JuCo III hat sich insgesamt der Anteil derjenigen, die sich einsam fühlen, leicht erhöht (36,5 % gegenüber in JuCo II 35,2 %). Es gibt Verschiebungen in den Gruppen, allerdings muss auch die große Ungleichheit der Fallzahlen berücksichtigt werden. Die größten Zuwächse sind bei jungen Menschen, die mit ihren Familien, in Jugend-WGs oder in einer Partnerschaft leben, zu verzeichnen.

Gesellschaftliche Institutionen und der öffentliche Raum sind als wesentliche Interaktionsorte für junge Menschen weggefallen. Zum Zeitpunkt der Durchführung von JuCo II wurde bereits wieder ein Lockdown verhängt. Aber auch in Zeiten sog. Lockerungen gab es Auflagen und, wie an den Studiendaten der Studie JuCo II zum Freizeitverhalten gezeigt werden kann, auch trotz vorhandener Möglichkeiten weniger Aktivitäten im Freizeitbereich (Andresen et al. 2021). Somit reduzieren sich soziale Kontakte stärker auf Bildungsinstitutionen bzw. den Arbeitsplatz. Diese institutionalisierten Kontakte haben offenkundig Einfluss auf das Einsamkeitserleben. So beschreiben sich diejenigen, die zur Schule (31 %), zur Arbeit gehen (29,4 %) oder eine Ausbildung machen (33,8 %), weniger oft als von Einsamkeit betroffen als diejenigen, die arbeitssuchend sind (41 %) oder studieren (44,9 %). Zu beiden letztgenannten Teilgruppen junger Menschen liegen bisher kaum fundierte Forschungsergebnisse zu psychischen Belastungen während der Pandemie vor (Traus et al. 2020; Besa et al. 2021).

**Figure 2:**
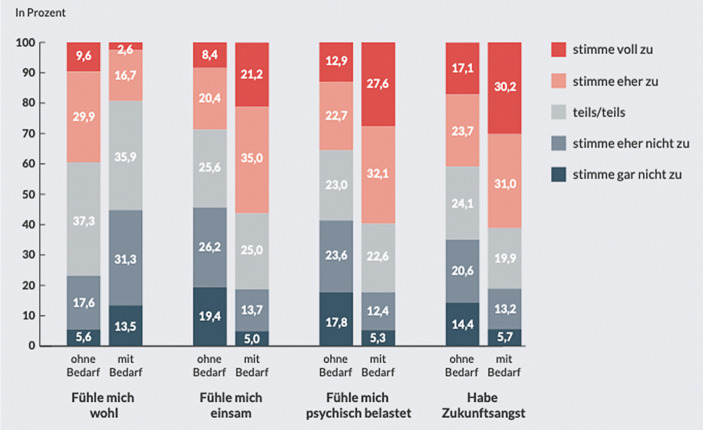

Aber die Verfügbarkeit öffentlicher Räume bzw. die Einschätzung junger Menschen dazu, wie ihnen diese zur Verfügung stehen, übt Einfluss auf das Erleben von Einsamkeit unter jungen Menschen während der Pandemie aus. Folgender Gruppenvergleich anhand der Daten aus JuCo II stellt heraus, dass junge Menschen, die über ihre private Wohn- und Lebenssituation hinaus einen Bedarf nach öffentlichen Orten „zum Abhängen“ haben, den sie zum Zeitpunkt der Befragung nicht erfüllt sehen, insgesamt stärker psychisch belastet sind und auch mit 56,2 % in dieser Gruppe überdurchschnittlich häufig Einsamkeit erleben als die Gruppe, die den Bedarf nach öffentlichen Räumen für sich nicht formuliert (28,8 %) (Abb. 2).

Junge Menschen, die Orte zum Abhängen vermissen, sei es auf öffentlichen Plätzen, in Freizeitstätten oder privat und unbeobachtet, befinden sich offensichtlich aktuell in einer besonders angespannten Lebenslage. Mit dem Wegfall der Orte für Begegnungen außerhalb des eigenen Haushalts erleben sie stärkere Belastungen als andere:

- 56,2 % von Ihnen fühlen sich einsam, 29,2 % geben dies von denjenigen an, die die öffentlichen Orte zum „Abhängen“ nicht vermissen.
- 44,8 % von Ihnen geben an, sich nicht wohlzufühlen, dies trifft auf nur 23,2 % derjenigen zu, die die öffentlichen Orte zum Abhängen nicht vermissen.

Auch in den Kategorien „ich fühle mich psychisch belastet“ und „ich habe Zukunftsangst“ fallen die Antworten derjenigen, die die öffentlichen Orte für sich als wichtige soziale Ressource vermissen, deutlich schlechter aus. Somit stellt sich Einsamkeit unter jungen Menschen auch stark über die fehlenden Gelegenheitsstrukturen für Begegnungen mit Peers her. Weiterhin sind junge Menschen mit Beeinträchtigungen in ihrer sozialen Teilhabe ohnehin eingeschränkt, auch stellt sich ihre Lebenssituation während der Corona-Pandemie noch schwieriger dar. 44,4 % der jungen Menschen, die in der Befragung JuCo II angaben, dass sie eine Beeinträchtigung haben und deshalb Unterstützung/Assistenz erhalten, gaben an, sich aktuell einsam zu fühlen. Weiterhin gab es eine Gruppe junger Menschen mit Beeinträchtigung, die auf nicht ausreichende Unterstützung zurückgreifen können. Unter ihnen gaben 57,4 % an, sich einsam zu fühlen.

**Figure 3:**
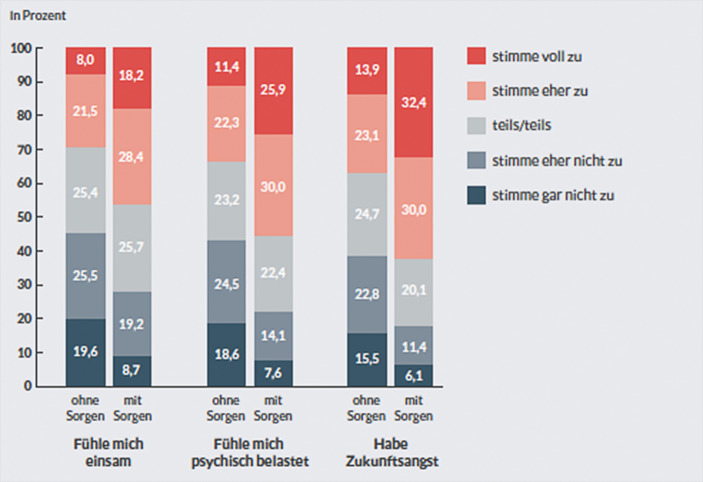

Das Einsamkeitsempfinden junger Menschen, so wird an folgender Grafik deutlich, variiert zudem stark je nach der ökonomischen Absicherung. Die jungen Menschen mit finanziellen Sorgen fühlen sich zu 46,6 % auch einsam. 62,4 % von ihnen haben Zukunftssorgen (Abb. 3).

Die Corona-Pandemie ist für die junge Generation kein punktuelles Ereignis, sie trifft dabei vor allem diejenigen jungen Menschen in besonderer Härte, die auch vorher schon sozial benachteiligt waren und über weniger soziale Ressourcen verfügen.

## Diskussion: Einsamkeit im Jugendalter – eine unterschätzte Kategorie für Wohlbefinden und soziale Ungleichheit

Einsamkeitserfahrungen haben, so konnte mit den JuCo-Studien gezeigt werden, während der Pandemie besonders an Bedeutung gewonnen. Die hohe Betroffenheit unter jungen Menschen ist unterschätzt worden, weil diese Thematik bisher kaum mit der Lebensphase von Kindheit und Jugend in Verbindung gebracht wurde. Es dominieren in Deutschland und international immer noch die Forschungen und Fachdiskurse zu Einsamkeit im höheren Lebensalter (Luhmann und Bücker 2019; Luhmann und Hawkley 2016; Hawkley et al. 2008; Huxhold und Engstler 2019). Entsprechend wenig Forschungsbefunde sind in diesem Themenfeld bisher verfügbar. Anhand der vorgelegten Ergebnisse konnte herausgearbeitet werden, dass Einsamkeit unter jungen Menschen während der Pandemie auch im Zusammenleben mit Familie oder anderen Personen ausgeprägt ist. Dieser Aspekt ist von besonderer Relevanz, da zwar bisher differenziert wird zwischen Alleinsein und Einsamkeit, hier also insbesondere die Lebensformen Alleinstehender differenziert analysiert werden. Gleichzeitig besteht bisher wenig Sensibilität für Einsamkeitserfahrungen von Menschen, die sich in natürlichen sozialen Gruppen bewegen und dort regelmäßig soziale Kontakte pflegen, sich dennoch nicht unbedingt zugehörig und aufgehoben fühlen. Entsprechend ist Einsamkeit in Familien, insbesondere in Krisen- oder Konfliktsituationen z. B. bei Trennung- und Scheidung oder in Alleinerziehenden-Haushalten (Nowland et al. 2021), bisher kaum erforscht.

Die Corona-Pandemie hat Lebensumstände hervorgebracht, in denen Begegnungen und soziale Aktivitäten stärker als bisher reglementiert werden und sich alternativ vermehrt in den digitalen Raum verlagern. Die Aktivitäten junger Menschen in einer zunehmend digitalisierten Lebenswelt bedeuten aber nicht, dass ihr soziales Leben ohne die physische Welt auskommt. Gleichzeitig gilt die Pandemie als eine Verstärkung von sozialen Problemen. So wird auch Einsamkeit zu einer gravierenden Begleiterscheinung, welche aber bereits vor Eintreten der Pandemie nicht besonders im Fokus sozialer Dienste, jugendpolitischer Forderungen oder wissenschaftlicher Forschung stand. Zwar sind die JuCo-Studien nicht repräsentativ, aber die große Fallzahl in allen drei Erhebungen sowie die umfängliche Mitteilungsbereitschaft bieten vertieften Einblick in die Lebenssituation junger Menschen anhand der quantitativen Befunde und der qualitativen Daten aus den Freitextfeldern des Erhebungsinstruments.

Es stellt sich die Frage, inwieweit junge Menschen, auch ungeachtet der Pandemie, von Einsamkeit und sozialen Ausschlüssen betroffen sind und welche Einflüsse diese Erfahrungen begünstigen. Es bedarf weitergehender Forschung, die über die Zeit der Pandemie hinausreicht, um vereinsamende Lebensbedingungen genauer zu identifizieren und insbesondere aus den Institutionen heraus, die junge Menschen adressieren, Konzepte für eine bessere Integration und Teilhabe in diesen Institutionen (wie Schule, Jugend- und Freizeitstätten) und durch diese zu ermöglichen. Dies muss zukünftig viel stärker auch eine digitale Strategie gegen Vereinsamung im Jugendalter mit einschließen. In den bisherigen Forschungsarbeiten zu Einsamkeit wird diese zwar auch in Zusammenhang mit deprivierten Lebenslagen gestellt (Stallberg 2021), die Lebenslagen von einsamen jungen Menschen kommen dabei aber bisher kaum vor, obwohl, so die Befunde der JuCo-Studien Einsamkeit unter jungen Menschen kein Randphänomen ist.

## Footnotes

1 Eurofound: European Quality of Life Survey 2016: https://www.eurofound.europa.eu/surveys/european-quality-of-life-surveys/european-quality-of-life-survey-2016.

2 Life with Corona Survey 2020: https://lifewithcorona.org.
